# Longitudinal course of behavioural and psychological symptoms of dementia: systematic review

**DOI:** 10.1192/bjp.bp.114.148403

**Published:** 2016-11

**Authors:** Rianne M. van der Linde, Tom Dening, Blossom C. M. Stephan, A. Matthew Prina, Elizabeth Evans, Carol Brayne

**Affiliations:** **Rianne M. van der Linde**, PhD, Institute of Public Health, University of Cambridge; **Tom Dening**, FRCPsych, Institute of Mental Health, University of Nottingham; **Blossom C. M. Stephan**, PhD, Institute of Health and Society, Newcastle University; **A. Matthew Prina**, PhD, Institute of Psychiatry, King's College London; **Elizabeth Evans**, PhD, Institute of Health and Society, Newcastle University; **Carol Brayne**, MD, Institute of Public Health, University of Cambridge, UK

## Abstract

**Background**

More information about the pattern of behavioural and psychological symptoms of dementia (BPSD) in the course of dementia is needed to inform patients and clinicians and to design future interventions.

**Aims**

To determine the persistence and incidence of BPSD and their relation to cognitive function, in individuals with dementia or in cohorts investigated for dementia onset.

**Method**

A systematic literature review analysed the baseline prevalence, persistence and incidence of 11 symptoms. The review was conducted according to established guidelines with the exception that we could not exclude the possibilities of bias in the studies examined.

**Results**

The 59 included studies showed considerable heterogeneity in their objectives and methods. The symptoms hyperactivity and apathy showed high persistence and incidence; depression and anxiety low or moderate persistence and moderate incidence; and psychotic symptoms low persistence with moderate or low incidence.

**Conclusions**

Despite heterogeneity across studies in terms of setting, focus and length of follow-up, there were clinically relevant differences in the longitudinal courses of different BPSD. Apathy was the only symptom with high baseline prevalence, persistence and incidence during the course of dementia.

Behavioural and psychological symptoms of dementia (BPSD) include affective symptoms, psychotic symptoms, non-aggressive agitation, irritability, wandering, elation and sleep problems.^1^ They have a high prevalence in dementia and nearly all people with dementia have at least one of these symptoms during the course of the disease.^2^ Such symptoms have negative effects on the quality of life of both patients and caregivers and are associated with increased costs of care.^3,4^ Better treatment and management of the symptoms are important, particularly as there is no effective treatment to alter the course of the underlying cognitive and functional decline. In order to design and conduct clinical trials for the treatment of BPSD, more information about the pattern of these symptoms in the different stages of dementia is needed to identify the best stage to intervene. In addition, insights into the extent to which BPSD occur over the course of dementia will help patients and care providers to plan for the future. Cross-sectional studies have shown that BPSD can occur at any time during the development of dementia. Their prevalence may increase from mild to severe dementia, whereas other studies suggest a non-linear course with the highest prevalence seen in the intermediate stages of disease.^5,6^ Symptoms may persist or be episodic over time, and this may differ between symptoms. Evidence from longitudinal studies is limited and has not been brought together systematically. Two reviews on the course of BPSD specifically in care-home residents have been published recently.^7,8^ They included a small number of studies (28 and 18) and concluded that the course of BPSD varied considerably between studies and between individual symptoms.

Our aim was to determine the longitudinal course of BPSD in individuals with dementia or in cohorts studied for dementia onset. We also investigated the persistence and incidence of symptoms and how persistence of BPSD over time relates to cognitive function. This review builds on five previous reviews by some of the same authors.^9–13^

## Method

Studies were eligible for inclusion if they reported the persistence, incidence or association with cognitive function of one or more BPSD in older adults (i.e. majority of participants aged at least 65 years) with dementia or cognitive impairment, and measured symptoms at three or more time points. Observational studies or intervention studies where there was a control group were included. The symptoms included were apathy, depressive symptoms, anxiety, irritability or aggression, non-aggressive agitation, hallucination, delusion, misidentification, sleep problems, wandering and elation. No language restriction was applied. Studies with inadequate descriptions of the sampling of the population or measurement of BPSD were excluded. The review protocol was not registered.

### Search method

Electronic searches of PubMed, EMBASE, Cinahl and PsycINFO databases were undertaken to identify potentially relevant articles published before March 2013. Search terms included text and MeSH terms for BPSD, dementia and longitudinal study (see online Fig. DS1). Two authors (R.v.d.L. and B.S.) independently searched titles and abstracts for potentially relevant articles. Following this, full text selection was completed by two authors: R.v.d.L. and A.M.P. (or B.S.). References of included studies were searched backwards and forwards, using the literature database Scopus.

### Data synthesis

Data were extracted independently and in duplicate (R.v.d.L. and B.S. or E.E.). Details extracted from each paper included setting, participant recruitment method, number of participants, follow-up time, BPSD and their measurement, number of BPSD measurements, population age (mean and range), baseline Mini-Mental State Examination (MMSE) score,^14^ baseline BPSD prevalence, statistical methods used, covariates taken into account and findings on the persistence, incidence and association of BPSD with cognitive function. Risk of bias was not formally assessed in a quality assessment. Findings were divided by dementia severity and BPSD. Dementia severity was defined using MMSE categories based on clinical practice guidelines from the National Institute for Health and Care Excellence (NICE): mild dementia (MMSE score 21–26), moderate dementia (MMSE 15–20), moderately severe dementia (MMSE 10–14) and severe dementia (MMSE <10).^15^ When no MMSE score was reported, equivalent cut-off scores from the Cambridge Cognitive Examination (CAMCOG), modified MMSE, Clinical Dementia Rating (CDR) scale and the Alzheimer's Disease Assessment Scale (ADAS) were used.^16–22^ By use of results from factor analyses and cluster analysis reported in the literature,^10^ symptoms were grouped into affective symptoms (comprising depression, anxiety and apathy), psychosis (comprising delusions and hallucinations), hyperactivity (comprising irritability, agitation and wandering), elation and sleep problems.

Where possible the persistence of symptoms was reported as the percentage of people with a certain symptom at baseline who also had the symptom at the next measurement or for whom the symptom persisted during the entire follow-up period. Incidence was reported as the percentage of people without symptoms at baseline who had developed new symptoms at the next measurement or during the entire follow-up period. Results from a multistate model were reported when available. Studies investigating the association between BPSD and cognitive function were summarised by reporting the analysis methods (e.g. Cox proportional hazards model, latent class linear mixed model or logistic regression model), covariates taken into account, BPSD score and results, including hazard ratios, β coefficients and *F* or *P* values.

Baseline BPSD prevalence, persistence, incidence and association with cognitive impairment were compared for each of the symptoms in the studies that included several BPSD. Prevalence, persistence and incidence were summarised as ‘low’ if the majority of studies found that the results were lower than that of most of the other symptoms included, ‘high’ if the majority found that results were higher than for most of the other symptoms and ‘moderate’ if the results were intermediate or mixed. The range of the results was reported for each symptom.

## Results

### 

Owing to considerable heterogeneity in study objectives and methods, inclusion criteria were revised *post hoc*. The following exclusion criteria were added: a follow-up period of less than 3 months; reporting only the prevalence at different time points; symptom measurement through retrospective caregiver report (retrospective studies using medical records were included); and measuring pure major depression or clinical depression only. Studies reporting on minor depression or depressive symptoms only or depression as part of BPSD were included (as discussed in two previous publications).^9,11^ From 5923 identified articles 48 were selected for inclusion after the abstract and full-text selection stages. Cross-referencing resulted in an additional 11 studies. In total 59 studies were included.

#### Study design

Characteristics of the studies included are shown in Table 1 and online Table DS1. The majority of studies recruited participants from psychiatric services including memory and dementia clinics (31 studies out of 59). Participants in these studies typically had moderate dementia (16 studies) and a younger mean age (in 20 studies participants had a mean age below 75 years). Other studies recruited participants from the population (8 studies), primary care (7 studies) or institutional care (8 studies), or recruited volunteers from other settings (4 studies). One study retrospectively reviewed medical records. The 8 studies that reported on care-home residents mostly included older participants (mean age 75 years or over) with moderately severe or severe dementia. Studies recruiting participants without dementia (10 studies) were found for depression only and most of these recruited from the general population (6 studies). Overall, most studies were from the USA or Canada (27 studies) and Europe (29 studies). Follow-up times ranged from 3 months to 14 years: 8 studies had a follow-up period of 1 year or less, 24 studies a follow-up period of 1–5 years and 26 had a follow-up period of 5 years or more.

**Table 1:**
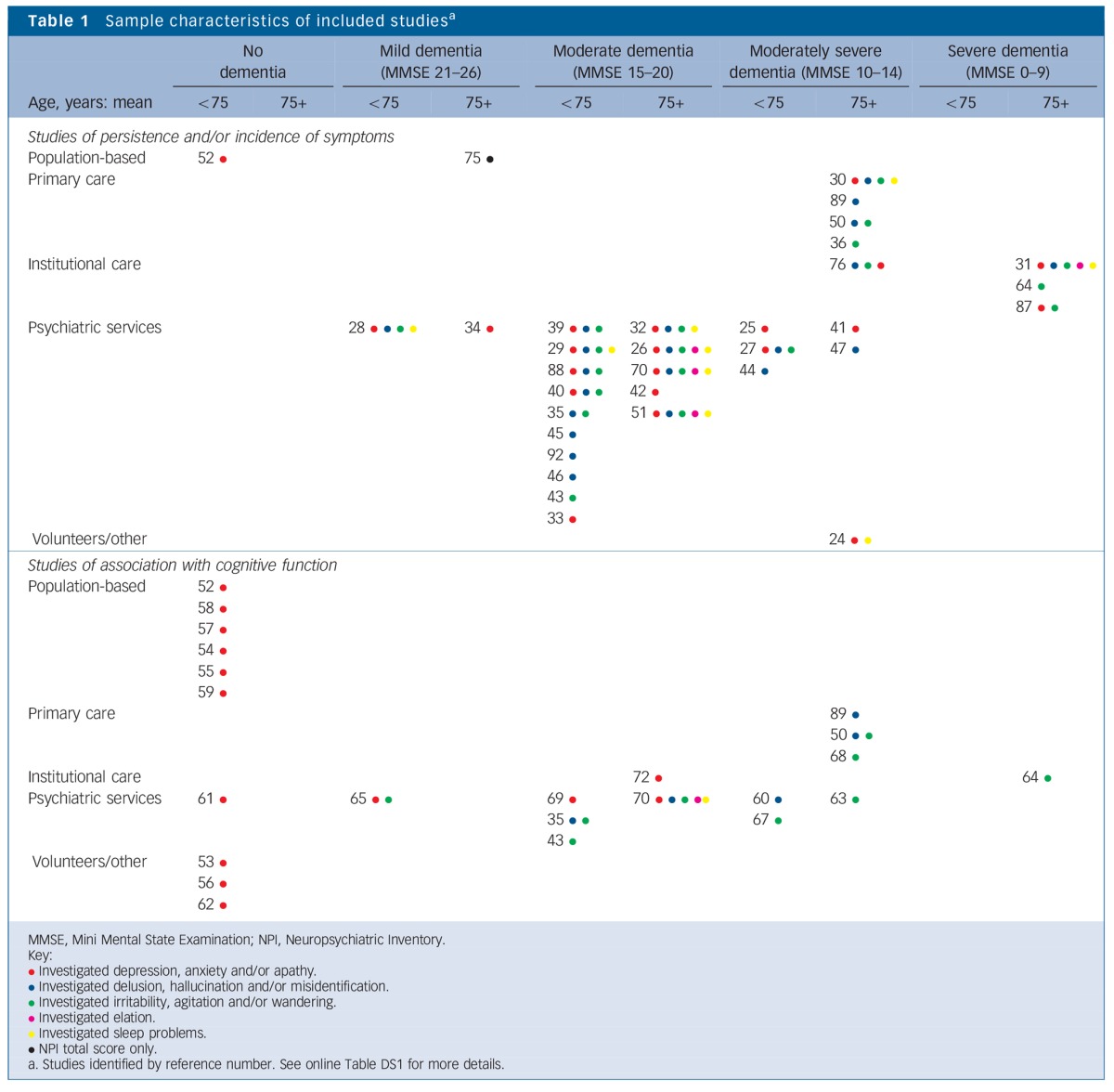
Sample characteristics of included studies^a^

### Symptoms

**Fig. 1:**
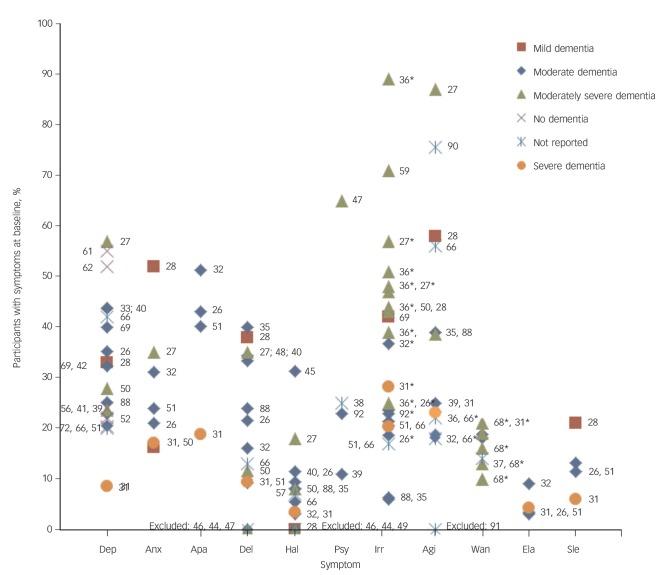
Baseline prevalence of behavioural and psychological symptoms; see online Table DS2 for more details. Numbers are the reference numbers of the included studies. ‘Excluded’ indicates that the study excluded participants with a particular symptom at baseline (i.e. the prevalence was 0%). Twenty-six studies that did not report baseline prevalence or reported on a population already included in the figure are omitted. Dep, depression; Anx, anxiety; Apa, apathy; Del, delusions; Hal, hallucinations; Psy, psychosis; Irr, irritability; Agi, agitation; Wan, wandering; Ela, elation; Sle, sleep problems. *Subsymptom reported separately.

Included symptoms are shown in Table 1 and their baseline prevalence is summarised in Fig. 1. Full details of each symptom, its definition, the instrument used for its measurement and the baseline prevalence can be found in online Table DS2. Affective symptoms were the most frequently studied (37 studies), with 24 studies reporting on depression only. Anxiety was studied in 11 studies, apathy in 4, and 2 studies reported on a factor of affective symptoms. Psychotic symptoms were studied in 26 studies (delusions in 20, hallucinations in 21, misidentifications in 1, psychosis symptoms combined in 5). Hyperactivity symptoms, including irritability (16 studies), non-aggressive agitation (often including pacing or wandering) (16 studies), wandering (4 studies) or a factor of hyperactivity symptoms (6 studies), were studied in 30 studies. Elation was measured in only 5 studies and sleep problems in 9. Many different instruments exist to measure and define BPSD,^12^ and 28 different instruments were used across the included studies. The Neuropsychiatric Inventory (NPI) was used in 8 studies.^23^ Five studies used the total score of a BPSD instrument, rather than presenting individual symptom profiles.

### Prevalence

The baseline prevalence varied widely across the studies (see Fig. 1). Generally, higher baseline prevalence was reported by studies that included a population with moderate or moderately severe dementia than by studies that included those with severe dementia only. A higher prevalence of symptoms was also seen in studies that recruited participants from psychiatric settings rather than from the population or institutional care settings (e.g. for depression: psychiatric settings 20–57%, institutional care 8–20%, population 22%). Studies with a younger mean age typically showed a higher symptom prevalence (e.g. for delusion: <75 years 24–40%, 75+ years 9–22%). There may also be differences by BPSD instrument. For example, studies that measured symptoms using the BEHAVE-AD typically showed a higher prevalence than studies using the NPI.

### Symptom persistence and remission

**Fig. 2:**
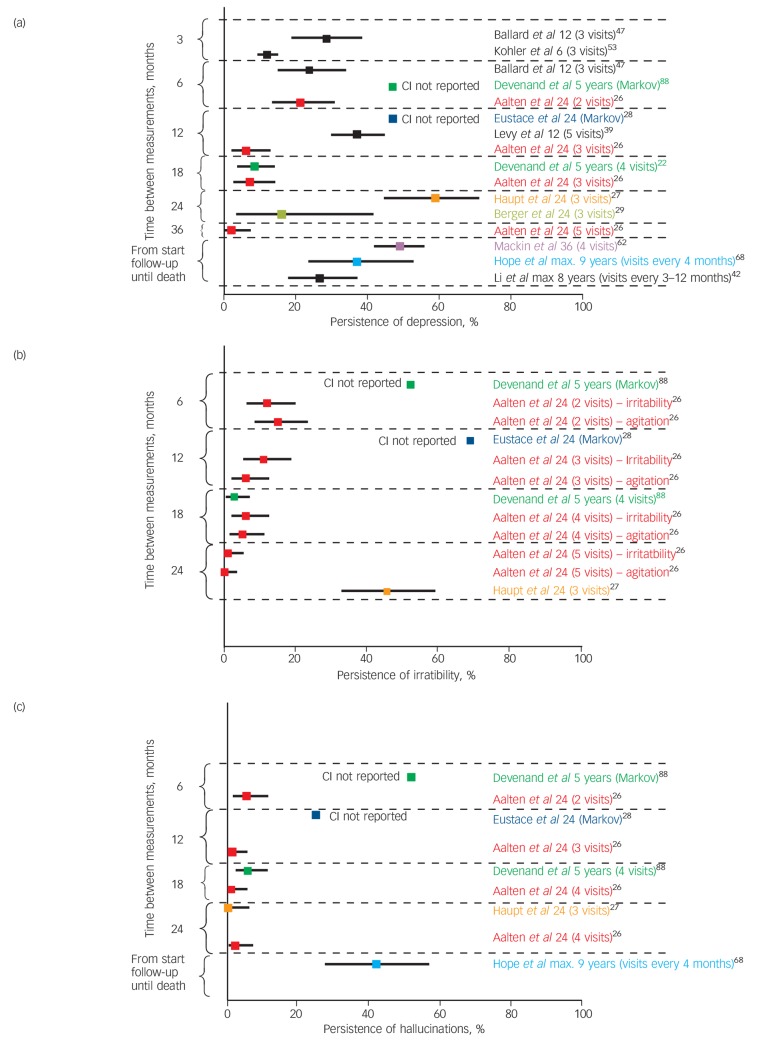
Persistence of (a) depression, (b) irritability and (c) hallucinations.Squares indicate the reported percentage where the symptom persisted over the measurement period and the lines indicate 95% confidence intervals. The name of the first author is given next to the corresponding findings. If the study reported the persistence over several intervals, it is included in the figure more than once. Next to the name of the author the total follow-up time (in months unless specified) and the number of visits are reported. For example, Aalten *et al* measured symptoms at 5 visits over 24 months and reported on the percentage of participants with depression present at any consecutive period of 6 months (depression present at 2 visits), 12 months (present at 3 visits), 18 months (present at 4 visits) or 24 months (present at 5 visits).

The persistence and stability of symptoms or the change in symptom scores over time were considered for each of the five symptom domains separately. Figure 2 summarises the results of studies investigating the persistence of depression, hallucination and irritability (see online Fig. DS2 for the persistence of all symptoms). Detailed findings are available in online Table DS3

#### Depression, anxiety and apathy

A large variation in persistence of affective symptoms was seen, with great intra-individual variability.^24,25^ Aalten *et al* reported a relatively low persistence of depression, anxiety and apathy,^26^ whereas Haupt *et al* reported a persistence of depression of up to 59% over a 2-year period.^27^ Anxiety and apathy may be more persistent over time than depressive symptoms,^26,28,29^ although two studies reported that anxiety was less persistent than depression.^27,30^ Wetzels *et al* (study not included in the figures because it described only the persistence over each observation) found that resolution of anxiety was consistently higher than persistence of symptoms, whereas apathy showed a variable course.^31^ Where change was modelled statistically, affective symptoms were generally found to be stable without significant change over time.^32–34^

#### Delusions, hallucinations and misidentifications

The persistence of psychotic symptoms was mostly below 30% (5 studies), although one study reported that the 6-month persistence of delusions was 59% and hallucinations 52%.^18^ Further, multistate models by Eustace *et al* showed delusions were persistent over 12 months in 65%.^28^ Generally, hallucinations were less persistent than symptoms of delusion,^18,26,28,35^ although one study found similar results for delusions and hallucinations,^27^ and two studies found hallucinations were more persistent than delusions.^30,31^

#### Irritability, agitation and wandering

Hyperactivity symptoms were mostly persistent, with one study showing that up to 76% of individuals had persistent symptoms of agitation over 2 years.^27^ Studies that investigated several hyperactivity symptoms found that agitation was more persistent than irritability.^18,26,27^ A study investigating several symptoms of irritability found that verbal aggression was the most common and longest-lasting form of aggressive behaviour, whereas aggressive resistance and physical aggression were most likely to persist until death.^36^ King-Kallimanis *et al* found that wandering status was more likely to change from wandering to non-wandering rather than the reverse and that wandering was a temporary phase for approximately half of care-home residents who were admitted as wanderers.^37^ Several authors analysed the course of hyperactivity over time using repeated measures analysis or a latent class linear mixed model. Garre-Olmo *et al* reported that over a 2-year period hyperactivity symptoms were mostly low and smoothly increasing (this pattern was found in two-thirds of participants).^32^ Cohen-Mansfield *et al* found that aggressive behaviours increased over time whereas physically non-aggressive behaviours did not change significantly.^38^

#### Elation

The persistence of elation was investigated in only two studies. Wetzels *et al* found in severe dementia that, for each two consecutive assessments at 0–6 months, 6–12 months, 12–18 months and 18–24 months, symptoms were stable in 39%, 18%, 3% and 3% respectively.^31^ Over a total follow-up period of 2 years Aalten *et al* found in moderate dementia that symptoms were stable over a 6-month period in 2%, whereas for none of the participants did symptoms persist over 12 months, 18 months or 24 months.^26^ Therefore, these results suggest that elation is not persistent.

#### Sleep problems

Most studies that investigated sleep problems (4 studies) reported low persistence,^26,29,30^ or a fluctuating course.^31^ In only one study were sleep symptoms reported to be persistent.^28^

### Incidence and absence of symptoms

**Fig. 3:**
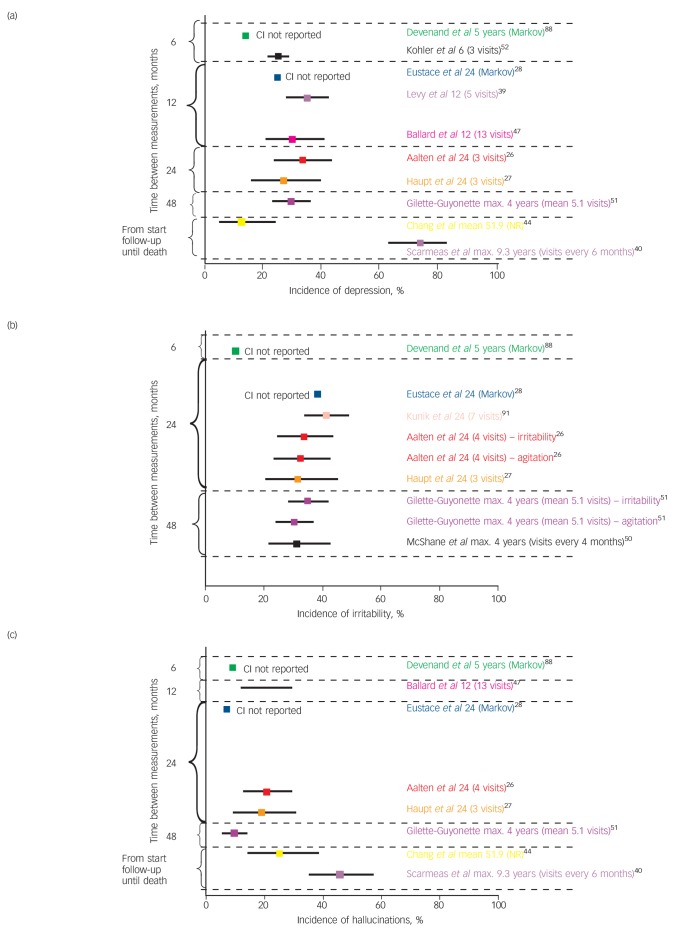
Incidence of (a) depression, (b) irritability and (c) hallucinations. See Fig. 2 for an explanation of the symbols. NR, not reported.

Online Table DS4 and Fig. DS3 show the incidence of symptoms and the percentage of participants who did not have symptoms during the follow-up period. A summary is shown in Fig. 3.

#### Depression, anxiety and apathy

Affective symptoms commonly develop in people with dementia. Over a 1-year period a high or moderate depression incidence of up to 37% was reported by eight studies,^26-28,31,39–42^ whereas in others the onset of depression was low compared with other symptoms.^18^ The incidence of apathy has been reported to be particularly high: 64% over 2 years,^26^ and 14–27% over a 6-month period.^31^

#### Delusions, hallucinations and misidentifications

In four studies the probability of new-onset hallucinations was reported to be low,^18,27,28,31^ whereas in another four incidence was reported to be moderate or high.^26,41,43,44^ Other psychotic symptoms including delusions showed a consistently moderate incidence (11 studies).^18,26,27,39,40,44–49^

#### Irritability, agitation and wandering

All included studies that compared the incidence of hyperactivity with other symptoms (9 studies) concluded that the incidence of hyperactivity was high or moderate.^18,26–28,31,39,40,50,51^ Although the incidence of agitation might be particularly high,^18,27,35^ wandering might develop less often.^37^

#### Elation

The incidence of elation was investigated by three studies that used the NPI. Aalten *et al* reported a cumulative incidence over a 2-year period in 5%,^26^ Wetzels *et al* reported that for each 6 months of observation new symptoms were seen in 3–4%,^31^ and Gillette-Guyonnet *et al* reported that new symptoms developed during a maximum follow-up of 4 years in 8%.^51^ These results suggest the incidence of elation is low.

#### Sleep problems

The probability of the onset of sleep problems was reported in four studies. No consistent findings were reported: at each 6-month period the incidence in one study was 15%,^28^ and in another 2–8%,^31^ whereas over a total follow-up of 2 years symptoms developed in 31%,^26^ and over 4 years in 11%.^51^

### Association with cognitive function

The results of studies investigating the association between the course of BPSD and cognitive function (25 studies) are summarised in online Table DS5.

#### BPSD and subsequent cognitive function

Eight studies investigated the association between depression and subsequent cognitive decline or development of dementia in those without dementia at baseline.^52–59^ Those with persistent depression showed significant decline over time in global cognitive function, memory, processing speed, recall and attention.^52,53,58^ Some found a slight increase in depression score before dementia diagnosis compared with those who did not develop dementia,^55,59^ whereas others did not find a significant change in depression before dementia diagnosis.^56,57^ In those with dementia, associations with progression of cognitive function were found for psychosis,^45,60^ hyperactivity,^43^ and depression.^33^ Two studies investigated the link between BPSD and mild cognitive impairment. In one study persistence of depression was associated with progression to dementia,^61^ whereas another reported no difference in persistence between those who were cognitively stable and those who progressed to dementia.^62^

#### Cognitive function and BPSD development

In individuals with dementia, psychosis, hyperactivity, agitation and physical aggression were associated with greater cognitive impairment.^26,35,38,63–65^ In contrast, Marin *et al* found no association in dementia between cognitive impairment and depression, delusion, agitation and irritability.^66^ Four studies found that symptoms increased with cognitive decline in the early stages of dementia and were most commonly seen in moderate dementia, followed by a declining or stable course in the final stages of dementia.^35,63,64,67^ Cognitive function at onset of wandering was found to differ by type of wandering behaviour; for example, results suggested that excessive walking was more common in mild dementia, whereas in severe dementia getting lost was more likely.^68^ No association was found between cognitive function and depressive symptoms in those with dementia,^69,70^ whereas in those without dementia and without depression at baseline, cognitive impairment at baseline was associated with an increase of depressive symptoms over time.^54^ In those aged 70 years and over cognitive function was found to be associated with initial scores for depression and anxiety, but not with symptom change over time.^71^ Baseline dementia diagnosis was not significantly associated with severity of depression at follow-up.^72^

### Comparison of symptoms

We summarised the studies investigating several BPSD to compare baseline prevalence, stability, incidence and association with cognitive function for each of the symptoms (Table 2). Some symptoms were studied more often than others, and evidence is lacking for infrequently studied symptoms such as wandering (included in only one study investigating several BPSD),^30^ and apathy and elation (included in only four studies).^26,31,32,51^ Depressive symptoms were most often studied (included in 12 of the 13 studies investigating several BPSD).^18,26–32,39,40,50,51^ Compared with other symptoms, the results suggest that the persistence and incidence of depressive symptoms are moderate. Anxiety seems to be less prevalent and was reported to have a moderate persistence and incidence.^26–31,51^ The few studies investigating apathy suggest a high prevalence, persistence and incidence of symptoms.^26,31,32,51^ The prevalence, persistence and incidence of psychotic symptoms were suggested to be low to moderate, and may be particularly low for hallucinations.^18,26–32,35,39,40,50,51^ Symptoms of hyperactivity were most frequently seen and the majority of studies reported a higher persistence and incidence compared with other symptoms.^18,26–29,31,32,35,39,40,50,51^

**Table 2:** Results of 13 studies reporting at least two behavioural and psychotic symptoms of dementia

Symptoms	Number of studies	Baseline prevalence (%) 11 studies	Persistence (%)^a^ 10 studies	Incidence (%)^b^ 9 studies
Affective	12	High	Moderate	Moderate
Depression	12	High (8–57%)	Moderate (16–70)	Moderate (10–73)
Anxiety	8	High (17–52%)	Moderate (17–52)	Moderate (12–38)
Apathy	4	High (19–51)	High (20–55)	High (27–64)

Psychosis	13	Low	Moderate	Moderate
Delusions	10	Moderate (9–40)	Low (0–82)	Moderate (5–84)
Hallucinations	11	Low (0–18)	Low (0–52)	Low (4–45)

Hyperactivity	12	High	High	High
Irritability	9	High (6–57)	Moderate (12–80)	High (10–69)
Agitation	7	High (18–87)	Moderate (21–77)	High (19–80)
Wandering	1	NR	High (60)	NR

Elation	4	Low (3–9)	Low (2–39)	Low (4–5)

Sleep problems	7	Moderate (6–11)	Low (10–57)	Low (8–31)

NR, not reported.

a. Percentage of symptoms persistent over 3 months or more.

b. Percentage incidence over 3 months or more.

## Discussion

This systematic review confirms that BPSD are common and relatively persistent in individuals with dementia. The results suggest there are differences between symptoms: hyperactivity and apathy showed high persistence and incidence; depression and anxiety low or moderate persistence and moderate incidence; and psychotic symptoms low persistence and a moderate or low incidence. Studies of the association between BPSD and cognitive function suggest that in those without dementia the presence of depression is associated with subsequent cognitive decline. In those with dementia, psychosis, hyperactivity, agitation and physical aggression were associated with greater cognitive impairment.

### Strengths and limitations

Standardised procedures were used for the literature search and data extraction, including double reading to ensure quality. However, no established search term for BPSD exists and therefore relevant studies may have been missed. The reference lists of included articles and reviews were searched to minimise the number of missed articles. The review included studies with a high degree of heterogeneity in study design and population characteristics, including large differences in the period over which the persistence and incidence was reported (1 month to 4 years), the total follow-up time (3 months to 14 years), the instrument and cut-off score used to measure symptoms, the number of symptoms measured, dementia severity, recruitment setting and mean age. This made cross-study comparisons difficult and a meta-analysis was not possible. We were not able to investigate whether the course of BPSD differs between types of dementia as only five of the 59 studies reported findings by dementia type. We adhered to most of the items of the Preferred Reporting Items for Systematic Reviews and Meta-Analyses (PRISMA) guidelines (see online Table DS6).^73^ Although we have reported on a range of factors that might influence the quality of the study, risk of bias was not formally assessed in a quality assessment. Our review therefore does not meet items 12, 15, 19 and 22 of the guidelines. Bias in the included studies may have led to an overestimate of persistence (e.g. participants remained under medical attention) or to an underestimate of persistence (e.g. gaps in the follow-up period or attrition through death or care-home admission). In addition, the review protocol was not registered.

#### Study differences

Many different instruments exist to measure BPSD,^74^ and 28 different instruments were used by the studies included in this review. However, the increasing use of the NPI might improve comparability of future studies.^13^ The NPI was used by eight studies. In these studies the baseline prevalence seemed lower than that reported by studies using other instruments (e.g. for irritability, NPI rates were 19–37%, Present Behavioural Examination (PBE)^93^ 25–89% and BEHAVE-AD^94^ 42–57%). The total score on the NPI significantly increased over time,^26,34,75^ and symptoms were mostly shown to be persistent,^26,31^ stable or increasing.^32,76^ The incidence reported by the studies using the NPI was low or moderate compared with studies using other instruments.^26,31,51^

Loss to follow-up is a challenge in longitudinal studies, and we have reported the number of participants at the end of the follow-up period for the included studies (online Table DS1). There was large variation in follow-up completion (24–100%, although often not reported) and reasons for leaving the study were often not reported. Persistence of BPSD may be associated with mortality and with refusal to participate in follow-up interviews, and differences in follow-up completion may have influenced the results. Furthermore, we have used study baseline as a proxy for disease and symptom onset and this may have affected the findings on the persistence of symptoms. Symptom course may be influenced by pharmacological or non-pharmacological interventions. Although there was large variation in medication use between study populations, in the majority of studies that included a sensitivity analysis the results were not altered when taking into account medication use. As we are not aware of a formal definition of high prevalence, persistence or incidence, we summarised the findings as ‘low’ if the majority of studies found that the results were lower than that of most of the other symptoms included, ‘high’ if the majority found that results were higher than for most of the other symptoms and ‘moderate’ if the results were intermediate or mixed.

### Interpretation of findings

#### Prevalence

The prevalence of symptoms varied across the studies and between symptoms. Depression, apathy, irritability, agitation and wandering showed a high prevalence, whereas the prevalence of anxiety, hallucination and elation was low. Some studies consistently reported a relatively low prevalence of symptoms,^18,26,31,66^ whereas others consistently reported a relatively high prevalence.^27,28,32^ Differences might be due to variability in study design, population characteristics or measurement of symptoms. Indeed, a higher prevalence of symptoms was generally seen in studies that recruited participants with less severe dementia, in studies that recruited from psychiatric settings rather than from the population or institutional care settings, and in studies with a younger mean age. There may also be differences due to the BPSD instrument used.

#### Persistence

Large differences in persistence were seen across symptom groups and individual symptoms. Affective symptoms (including depression, anxiety and apathy) generally showed a moderate persistence, although a limited number of studies reported persistence of apathy to be high and in one study it was reported to be higher than for any other symptom.^26^ Persistence of psychosis was low to moderate. In contrast, hyperactivity symptoms showed a high persistence. This is an issue of concern as these symptoms are among those most problematic for caregivers.^77,78^ A low persistence was seen for elation and sleep problems. Differences in symptom persistence may reflect the nature of the symptom or might be explained by factors such as more widely available treatment options for depression and anxiety. Differences in dementia severity and baseline BPSD prevalence are likely to have affected results on persistence of symptoms. Persistence may be higher in those with more severe cognitive impairment at baseline,^32,35,38,46^ and a higher BPSD prevalence.^32,39^ However, associations between study characteristics and results could not be tested because of the large degree of heterogeneity in study design and population characteristics.

#### Incidence

The results suggest that affective symptoms and hyperactivity symptoms commonly develop in people with dementia. Large differences in reported incidence were seen between studies. For example, the reported incidence of depression ranged from 12% over a mean follow-up period of 52 months,^44^ to 73% over a maximum follow-up period of 9.3 years.^40^ Differences in study design and differences in baseline prevalence of symptoms are likely to have influenced the results. For example, the reported incidence might be higher in studies that reported a high baseline prevalence,^27^ compared with studies with a low baseline prevalence,^18,28^ although no formal analysis of the association between study characteristics and incidence was possible.

### Role of cognition

The presence of depression before the onset of dementia was associated with subsequent cognitive decline.^52,53,58^ In dementia, psychosis, hyperactivity, agitation and physical aggression were associated with greater cognitive impairment.^35,38,63–65,70^ Symptoms may be most common in moderate dementia, followed by a declining or stable course in the final stages of dementia.^35,63,64^ However, heterogeneity in the pattern of findings across studies investigating the associations between BPSD and cognitive function prevented us from drawing more specific conclusions. The heterogeneity of results does, however, suggest that BPSD do not solely arise secondary to cognitive impairment.

### Study implications

The results from this systematic review suggest that some symptoms such as hyperactivity are more persistent than others such as elation and sleep problems. In particular apathy, irritability, agitation and wandering showed a high persistence. These symptoms should be targeted in clinical trials to improve management and intervention. Clinical trials typically follow participants with more severe dementia over a short period.^79,80^ However, results presented here show that symptoms may persist over long periods until death,^18,30,42^ and may be most common in moderate dementia.^35,63,64,67^ Clinical trials focusing on the earlier stages of dementia with a long follow-up time might therefore be particularly informative. Results could also inform patients and care providers about which symptoms are most likely to recur, so that measures can be put in place to reduce their impact. Recommendations for monitoring of patients and symptom management interventions are outlined in guidance by the Alzheimer's Society.^81^

### Future research

The heterogeneity in methods and results emphasises the importance of clearly reporting the study design, population characteristics and symptom definitions. Table 1 shows that studies typically included younger populations with moderate dementia, whereas studies recruiting those with mild or moderate dementia from the population or from primary care settings were lacking. As BPSD patterns may differ in these populations, they should be the focus of future studies. In addition, all included studies were conducted in high-income countries and the findings may therefore not be applicable outside these settings. Apathy was infrequently studied, and as the limited results suggest that it may have a high persistence and incidence, we recommend that this symptom should be the focus of future studies on symptom course.

These methodological issues reiterate the findings from several of our previous reviews. A review of reviews showed a focus on individual symptoms (particularly depression), raised the question how best to define and measure BPSD within and across populations, and recommended reporting more clearly the characteristics of the population, the inclusion and exclusion criteria and how BPSD were defined and measured.^9^ Two reviews concluded that there were many instruments to measure BPSD,^12,13^ of which the NPI – a short, informant-based questionnaire measuring ten symptoms – has been cited most frequently and should form the core of any battery, although researchers choosing instruments should carefully address any gaps in its content with regard to their research question. In a guest editorial we discussed that the populations used in studies of depression and BPSD are often not quite comparable and that the results therefore cannot be readily extrapolated.^11^ Finally, we showed that studies investigating symptom groups show relatively consistent results, although there remains a large amount of individual variability.^10^

Studying covariates that may be associated with higher persistence of BPSD, including impairment in activities of daily living,^27,32,64^ as well as medication use,^38,46^ could improve understanding of potential mechanisms involved in the presence and persistence of BPSD. Environmental factors such as overstimulation and a person's surroundings, as well as physical factors such as pain and dehydration, are recognised as important triggers for BPSD.^5,82–85^ These factors are often difficult to capture and have not been investigated in the studies included here.

### Clinical implications

Our findings underscore the existing evidence that BPSD are common in dementia and that they are also relatively persistent. Different symptoms have a variable course over time: for example, psychotic symptoms have relatively low persistence – that is, they may resolve during the course of the dementia. In contrast, apathy emerged as the only individual symptom with high baseline prevalence, high persistence and also a high incidence during the course of the dementia. Thus, increased interest in apathy as a possible early sign of dementia, as a marker for underlying brain changes and as a sign of progression of dementia seems entirely warranted.^86^ Although hyperactivity as a whole also had high baseline prevalence, high persistence and high incidence over time, the various symptoms subsumed under hyperactivity mean that it is not a unitary phenomenon. These findings are relevant to clinicians as they indicate which symptoms may be expected to persist or to occur anew, and therefore give a better understanding of the natural history of BPSD which, in turn, can influence approaches to management and treatment.
